# Supplementary material for: Chemosensory Receptor Expression in the Abdomen Tip of the Female Codling Moth, Cydia pomonella L. (Lepidoptera: Tortricidae)
Source: Insects. 2023 Dec 14;14(12):948. doi: 10.3390/insects14120948 (PMC10743790; doi:10.3390/insects14120948)
Supplement: Supplementary file 1 [file insects-14-00948-s001.zip › DataFile S5 - Combined All Uncropped Gel Images - RF.pdf]

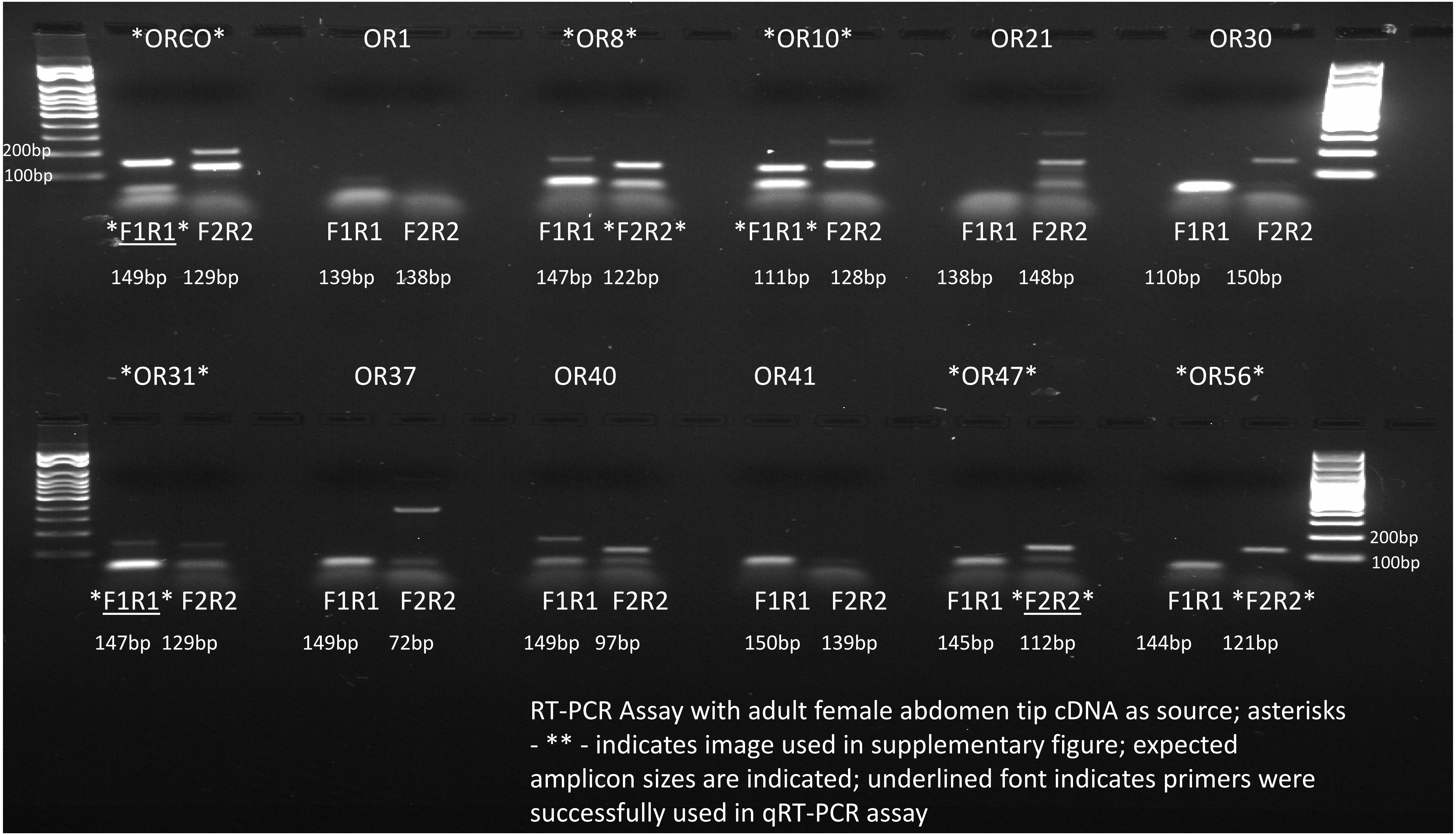

RT-PCR Assay with adult female abdomen tip cDNA as source; asterisks - ** - indicates image used in supplementary figure; expected amplicon sizes are indicated; underlined font indicates primers were successfully used in qRT-PCR assay

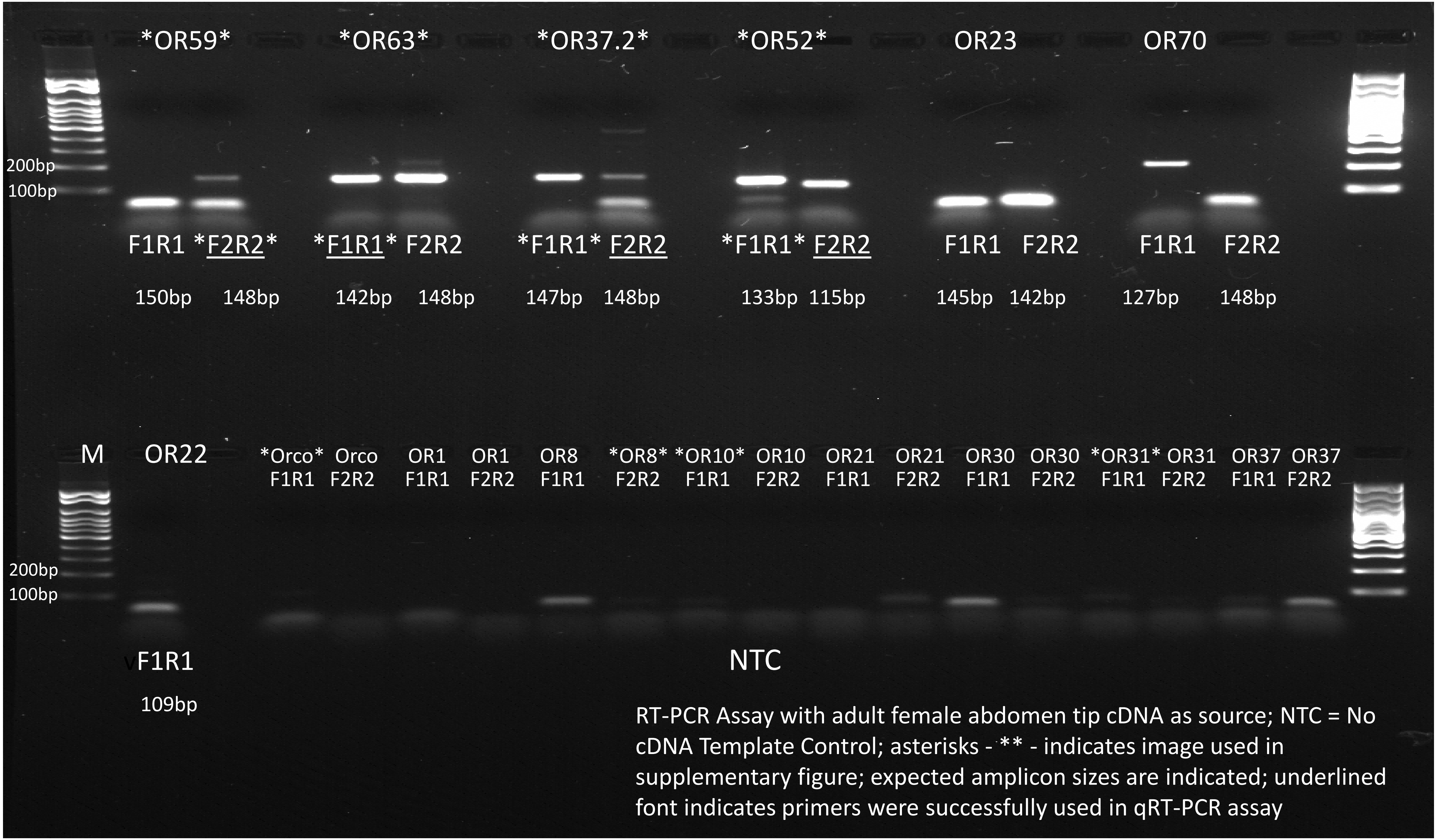
OR59
OR63
OR37.2
OR52
OR23
OR70
200bp
100bp
F1R1 *F2R2*
F1R1 F2R2
F1R1 F2R2
F1R1 F2R2
F1R1 F2R2
F1R1 F2R2
150bp 148bp
142bp 148bp
147bp 148bp
133bp 115bp
145bp 142bp
127bp 148bp
M
OR22
Orco F1R1
Orco F2R2
OR1 F1R1
OR1 F2R2
OR8 F1R1
OR8 F2R2
OR10 F1R1
OR10 F2R2
OR21 F1R1
OR21 F2R2
OR30 F1R1
OR30 F2R2
OR31 F1R1
OR31 F2R2
OR37 F1R1
OR37 F2R2
200bp
100bp
F1R1
109bp
NTC
RT-PCR Assay with adult female abdomen tip cDNA as source; NTC = No cDNA Template Control; asterisks - ** - indicates image used in supplementary figure; expected amplicon sizes are indicated; underlined font indicates primers were successfully used in qRT-PCR assay

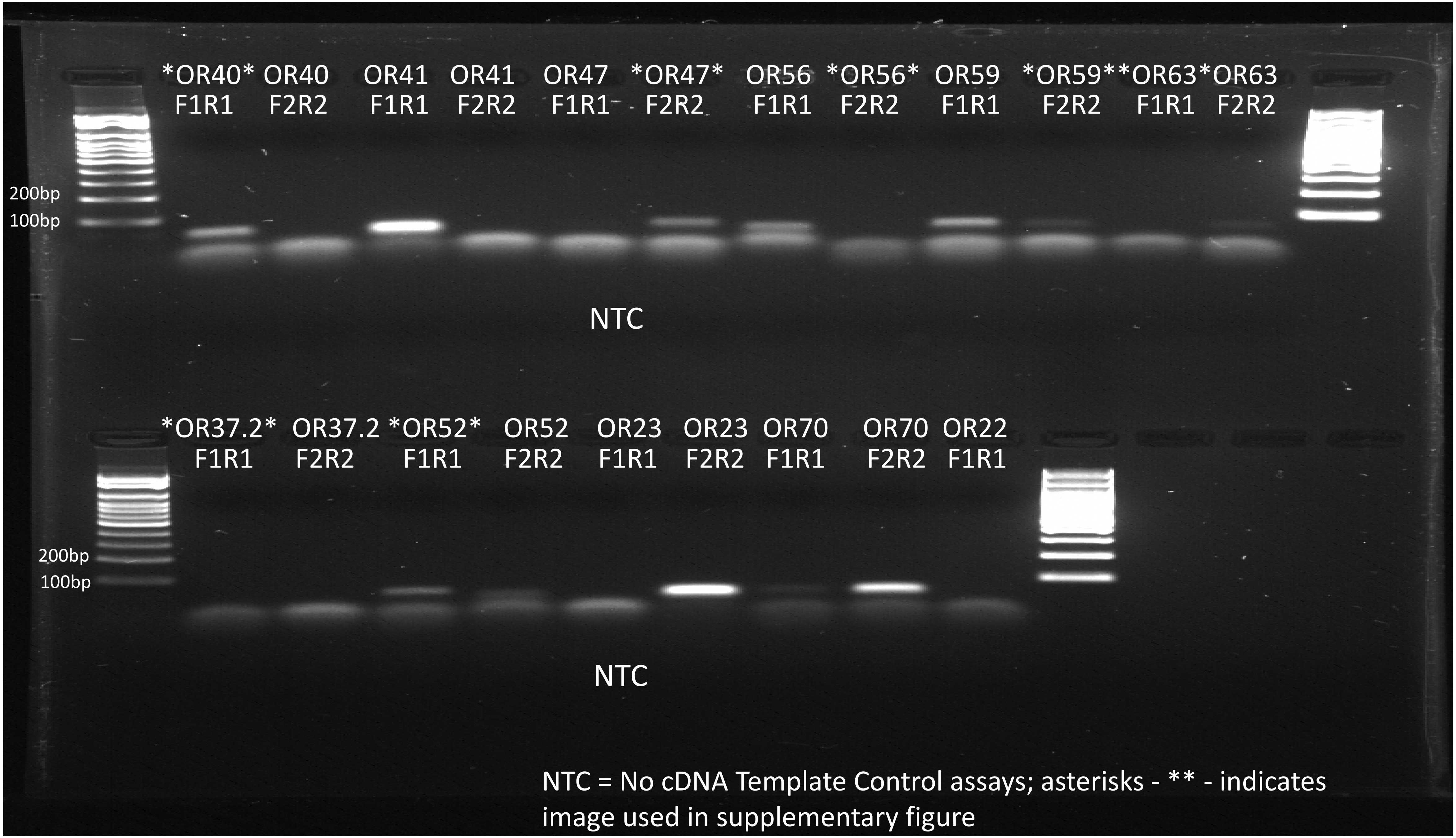
OR40 F1R1
OR40 F2R2
OR41 F1R1
OR41 F2R2
OR47 F1R1
OR47 F2R2
OR56 F1R1
OR56 F2R2
OR59 F1R1
OR59 F2R2
OR63 F1R1
OR63 F2R2
200bp
100bp
NTC
OR37.2 F1R1
OR37.2 F2R2
OR52 F1R1
OR52 F2R2
OR23 F1R1
OR23 F2R2
OR70 F1R1
OR70 F2R2
OR22 F1R1
200bp
100bp
NTC
NTC = No cDNA Template Control assays; asterisks - ** - indicates image used in supplementary figure

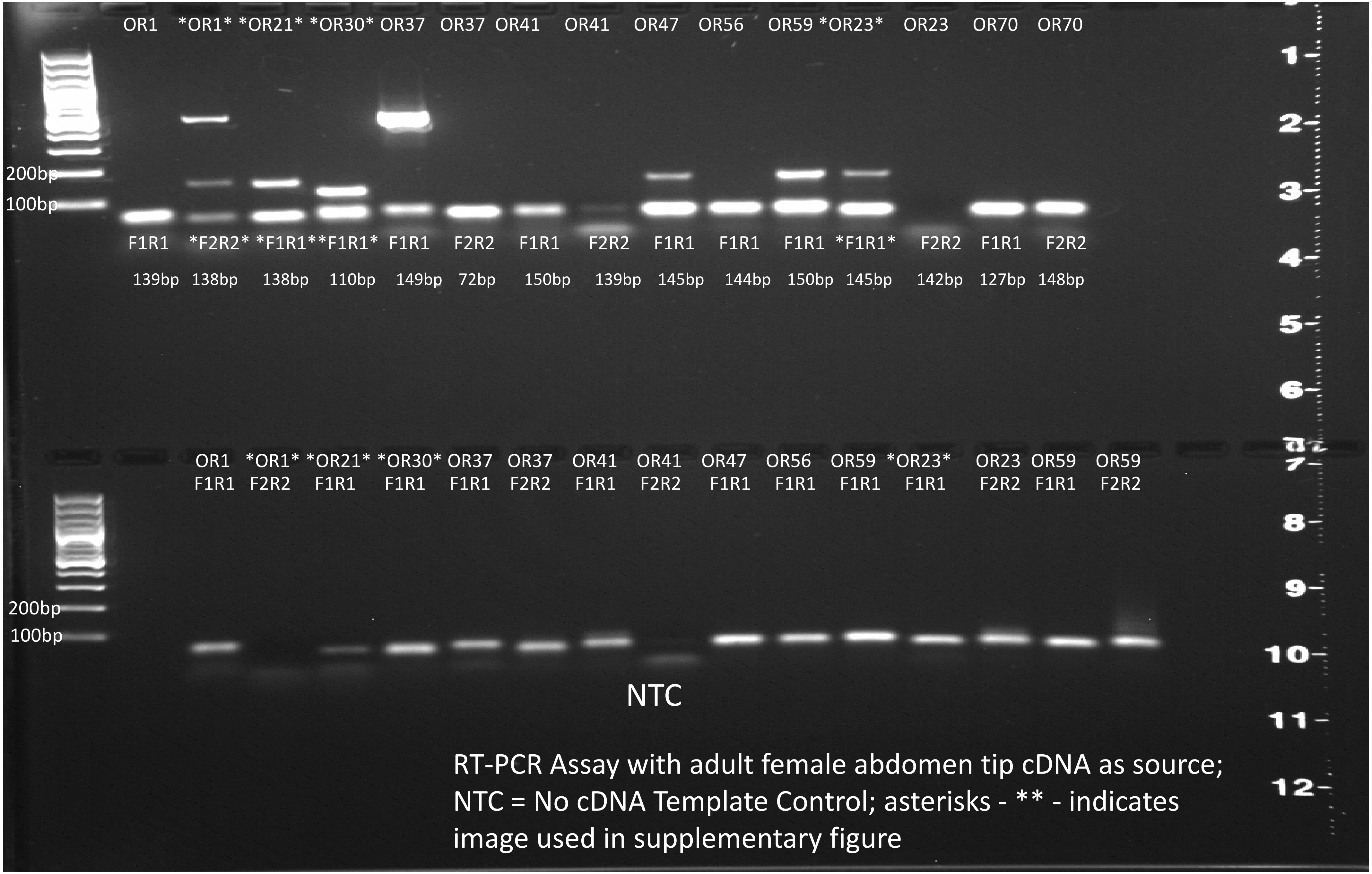
OR1 *OR1* *OR21* *OR30* OR37 OR37 OR41 OR41 OR47 OR56 OR59 *OR23* OR23 OR70 OR70
200bp
100bp
F1R1 *F2R2* *F1R1* *F1R1* F1R1 F2R2 F1R1 F2R2 F1R1 F1R1 F1R1 *F1R1* F2R2 F1R1 F2R2
139bp 138bp 138bp 110bp 149bp 72bp 150bp 139bp 145bp 144bp 150bp 145bp 142bp 127bp 148bp
OR1 F1R1
OR1 F2R2
OR21 F1R1
OR30 F1R1
OR37 F1R1
OR37 F2R2
OR41 F1R1
OR41 F2R2
OR47 F1R1
OR56 F1R1
OR59 F1R1
OR23 F1R1
OR23 F2R2
OR59 F1R1
OR59 F2R2
200bp
100bp
NTC
1
2
3
4
5
6
7
8
9
10
11
12
RT-PCR Assay with adult female abdomen tip cDNA as source;
NTC = No cDNA Template Control; asterisks - ** - indicates
image used in supplementary figure

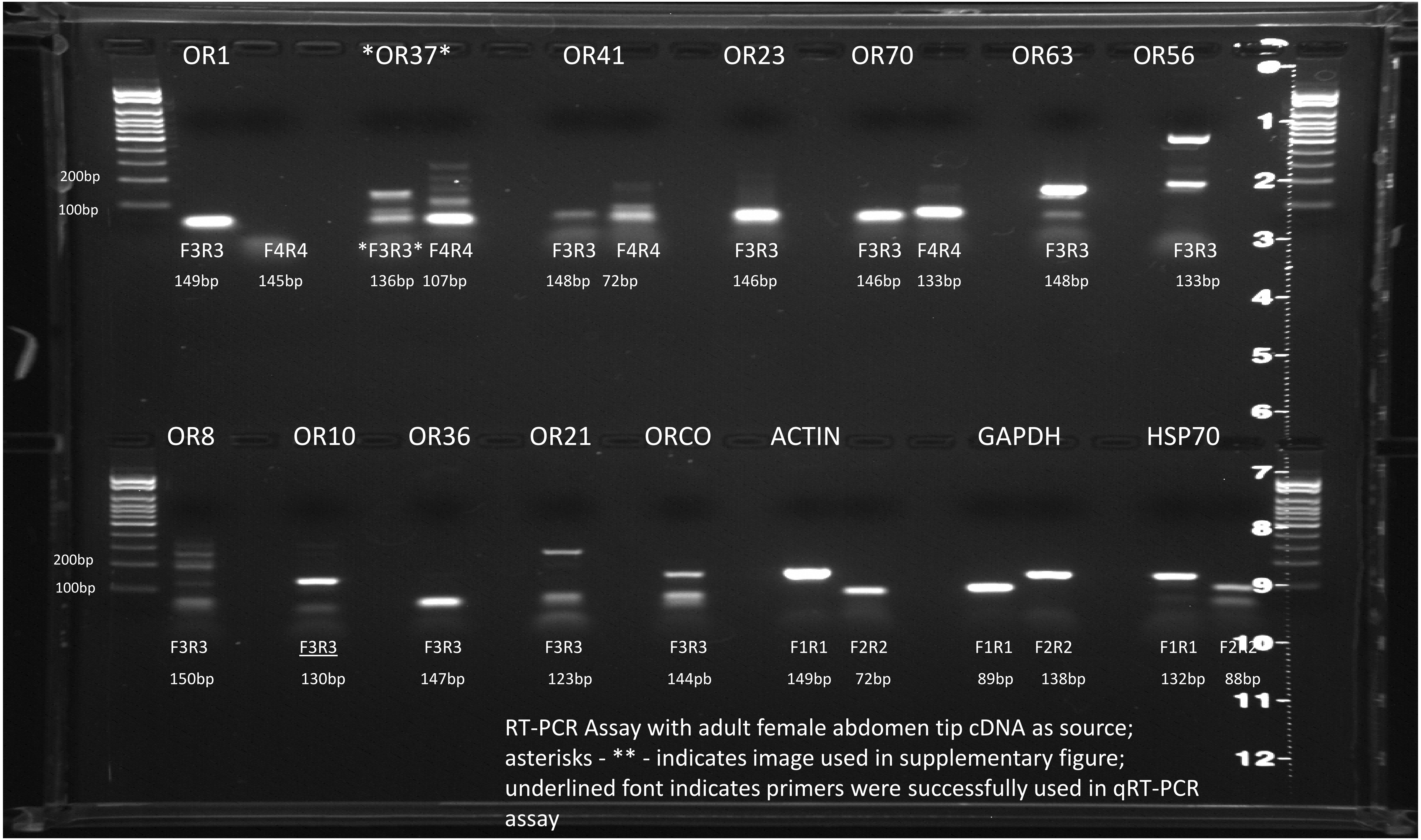
OR1
OR37
OR41
OR23
OR70
OR63
OR56
200bp
100bp
F3R3 F4R4 *F3R3* F4R4 F3R3 F4R4 F3R3 F3R3 F4R4 F3R3 F3R3
149bp 145bp 136bp 107bp 148bp 72bp 146bp 146bp 133bp 148bp 133bp
OR8
OR10
OR36
OR21
ORCO
ACTIN
GAPDH
HSP70
200bp
100bp
F3R3 F3R3 F3R3 F3R3 F3R3 F1R1 F2R2 F1R1 F2R2 F1R1
150bp 130bp 147bp 123bp 144pb 149bp 72bp 89bp 138bp 132bp 88bp
RT-PCR Assay with adult female abdomen tip cDNA as source;
asterisks - ** - indicates image used in supplementary figure;
underlined font indicates primers were successfully used in qRT-PCR
assay

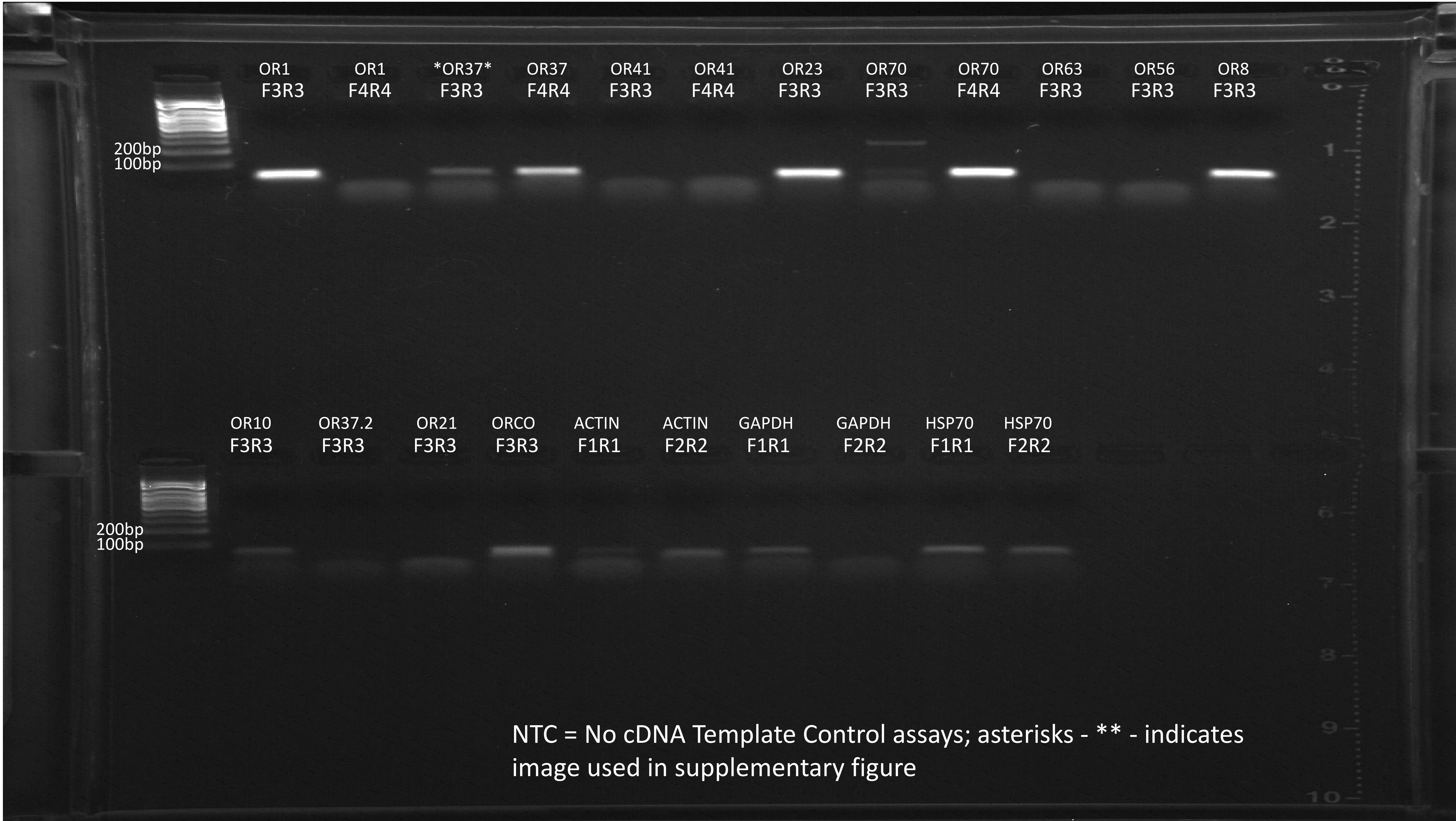
OR1 F3R3
OR1 F4R4
OR37 F3R3
OR37 F4R4
OR41 F3R3
OR41 F4R4
OR23 F3R3
OR70 F3R3
OR70 F4R4
OR63 F3R3
OR56 F3R3
OR8 F3R3
200bp
100bp
OR10 F3R3
OR37.2 F3R3
OR21 F3R3
ORCO F3R3
ACTIN F1R1
ACTIN F2R2
GAPDH F1R1
GAPDH F2R2
HSP70 F1R1
HSP70 F2R2
200bp
100bp
NTC = No cDNA Template Control assays; asterisks - ** - indicates image used in supplementary figure

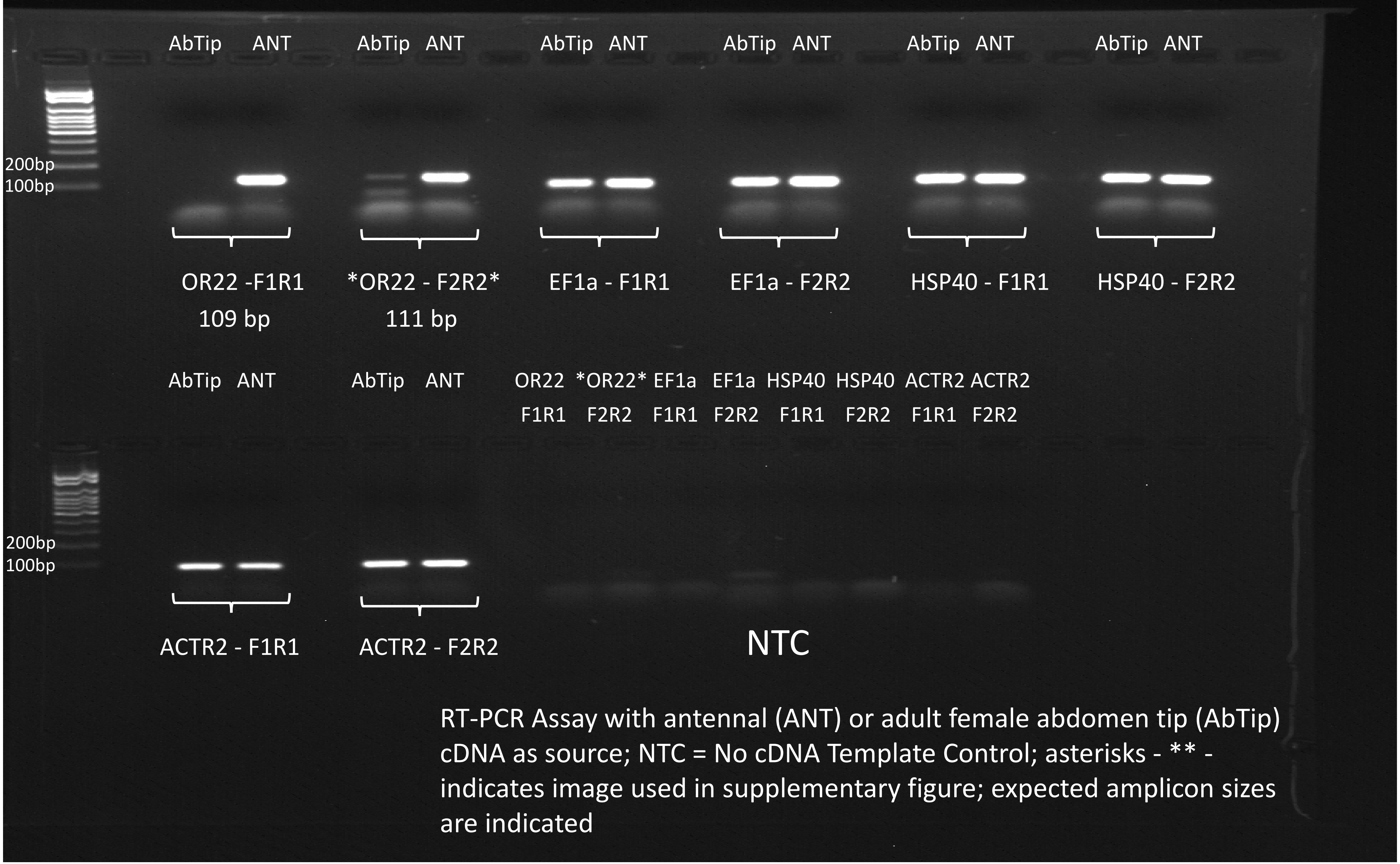

RT-PCR Assay with antennal (ANT) or adult female abdomen tip (AbTip) cDNA as source; NTC = No cDNA Template Control; asterisks - ** - indicates image used in supplementary figure; expected amplicon sizes are indicated

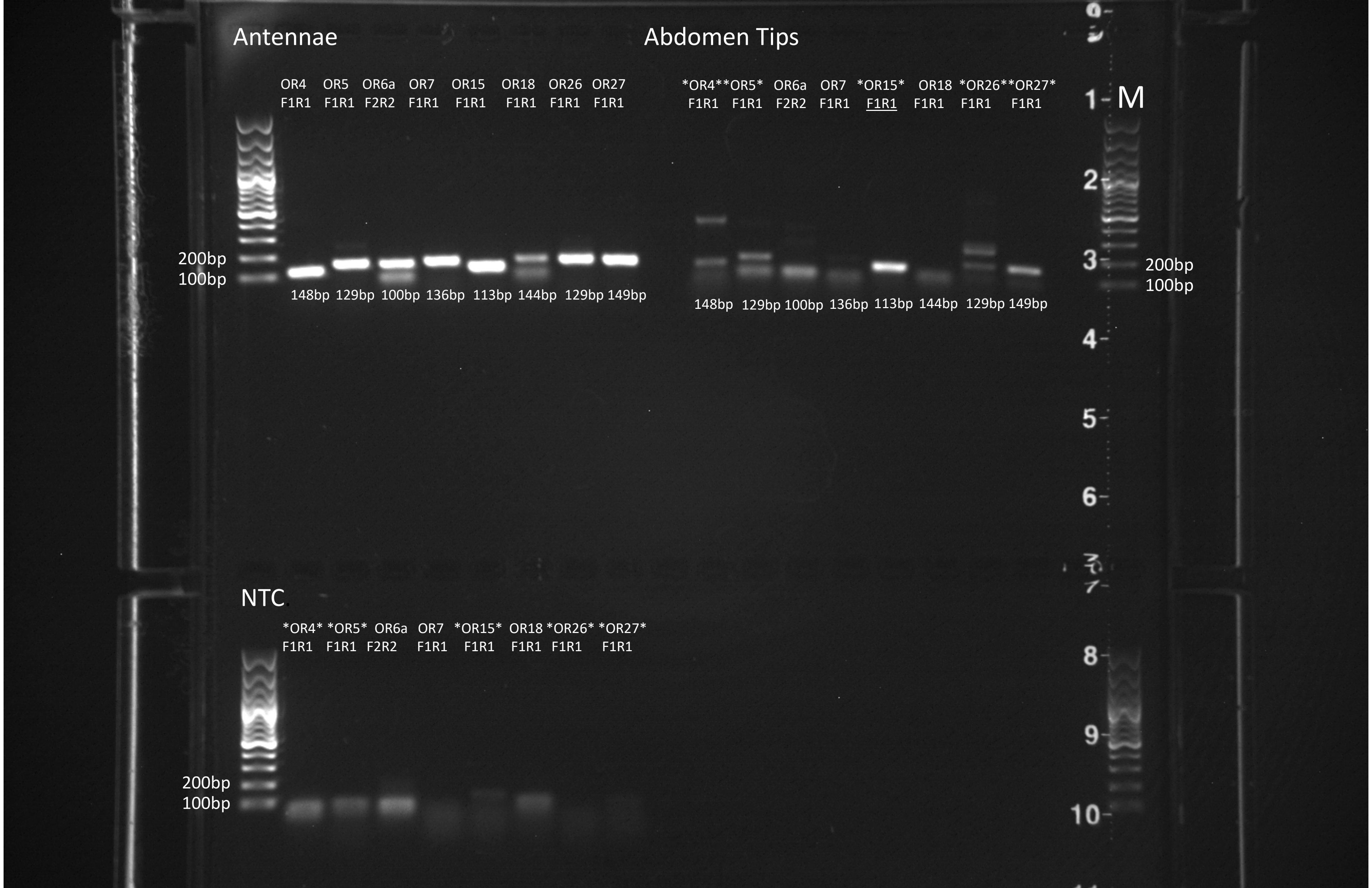

RT-PCR Assay with antennal or adult female abdomen tip cDNA as source; NTC = No cDNA Template Control; asterisks - ** - indicates image used in supplementary figure; expected amplicon sizes are indicated; underlined font indicates primers were successfully used in qRT-PCR assay

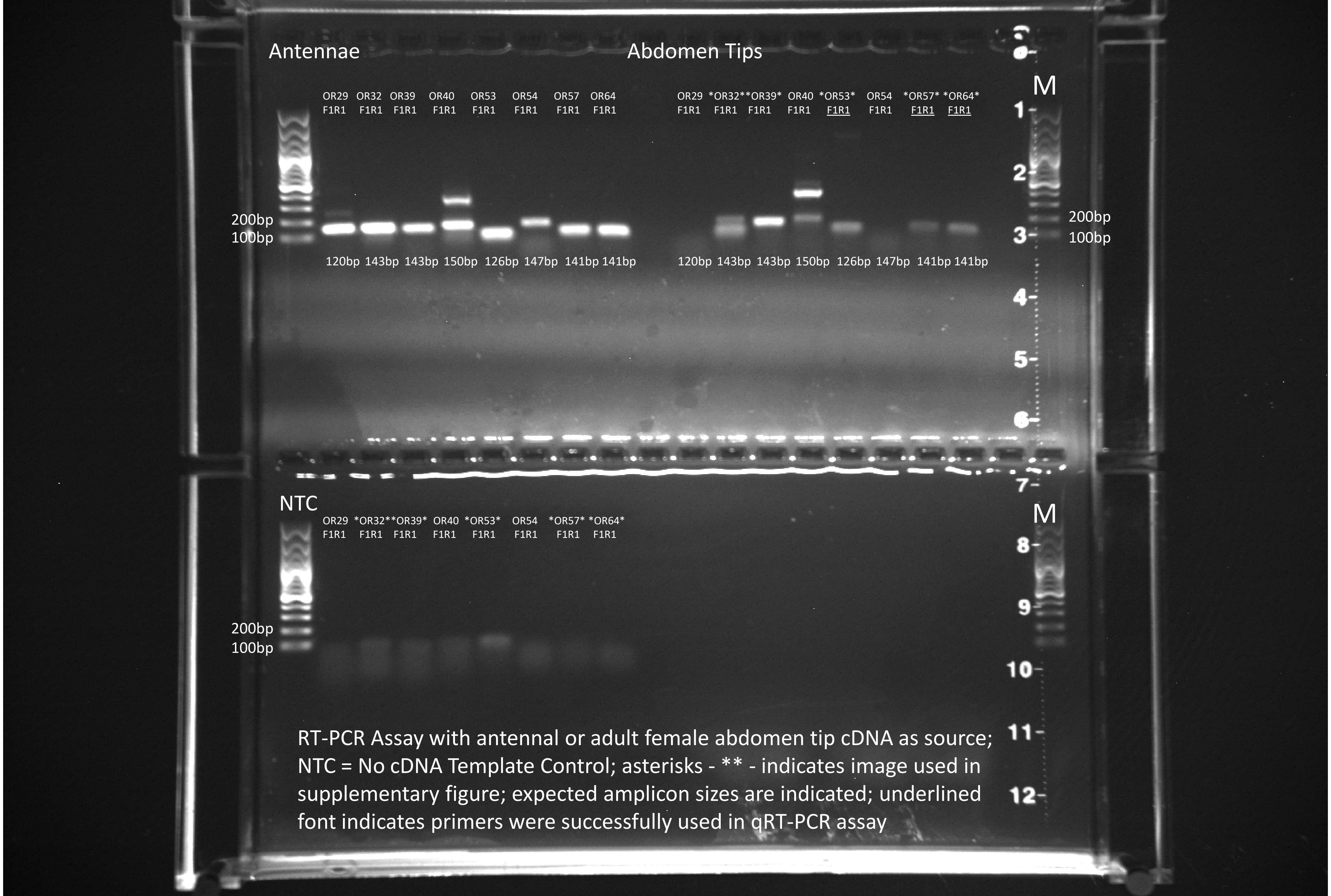
Antennae
Abdomen Tips
M
OR29 F1R1
OR32 F1R1
OR39 F1R1
OR40 F1R1
OR53 F1R1
OR54 F1R1
OR57 F1R1
OR64 F1R1
OR29 F1R1
*OR32** F1R1
OR39 F1R1
OR40 F1R1
OR53 F1R1
OR54 F1R1
OR57 F1R1
OR64 F1R1
200bp
100bp
120bp 143bp 143bp 150bp 126bp 147bp 141bp 141bp
120bp 143bp 143bp 150bp 126bp 147bp 141bp 141bp
NTC
OR29 F1R1
*OR32** F1R1
OR39 F1R1
OR40 F1R1
OR53 F1R1
OR54 F1R1
OR57 F1R1
OR64 F1R1
RT-PCR Assay with antennal or adult female abdomen tip cDNA as source; NTC = No cDNA Template Control; asterisks - ** - indicates image used in supplementary figure; expected amplicon sizes are indicated; underlined font indicates primers were successfully used in qRT-PCR assay

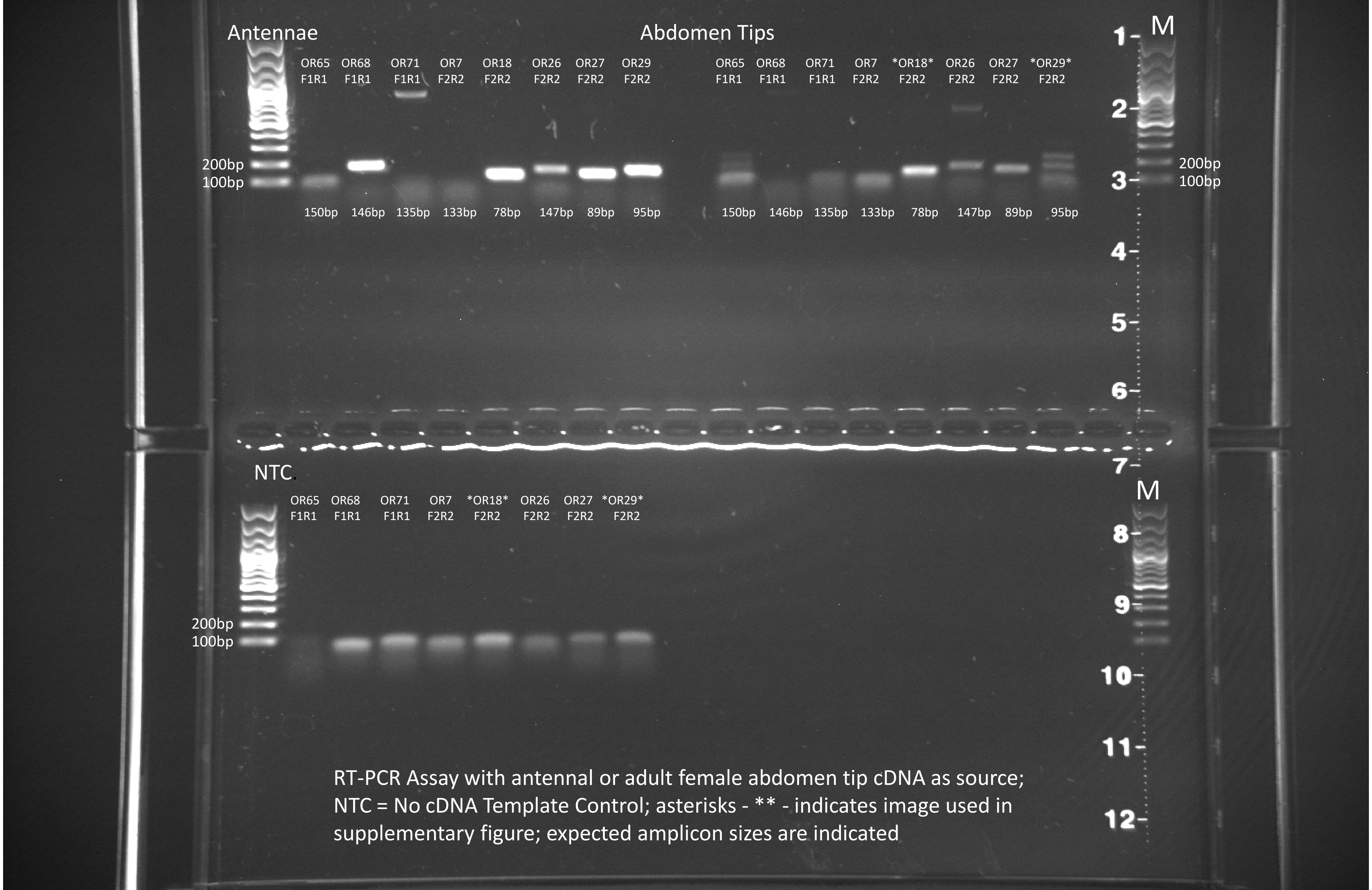

RT-PCR Assay with antennal or adult female abdomen tip cDNA as source; NTC = No cDNA Template Control; asterisks - ** - indicates image used in supplementary figure; expected amplicon sizes are indicated

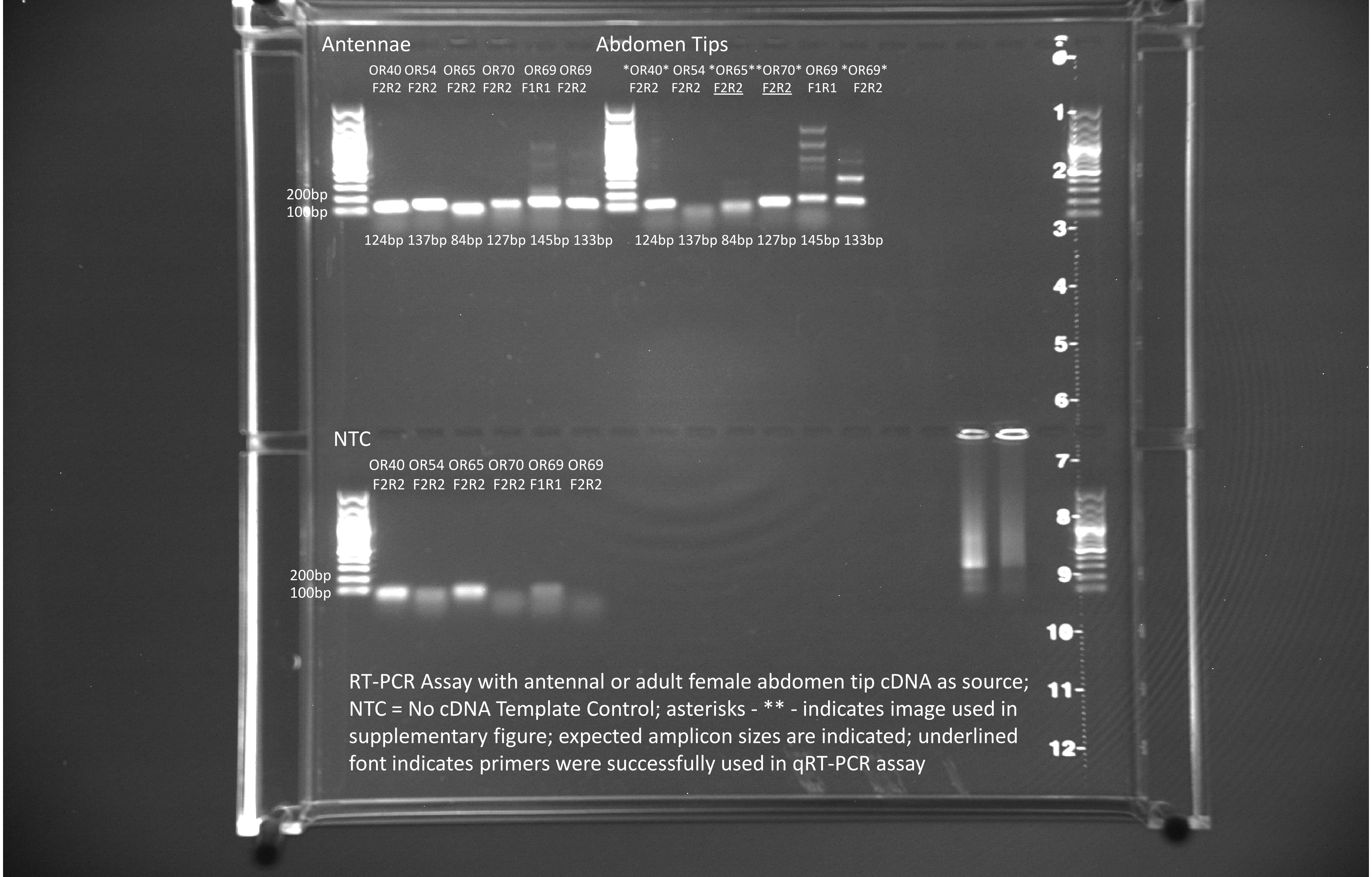

RT-PCR Assay with antennal or adult female abdomen tip cDNA as source; NTC = No cDNA Template Control; asterisks - ** - indicates image used in supplementary figure; expected amplicon sizes are indicated; underlined font indicates primers were successfully used in qRT-PCR assay

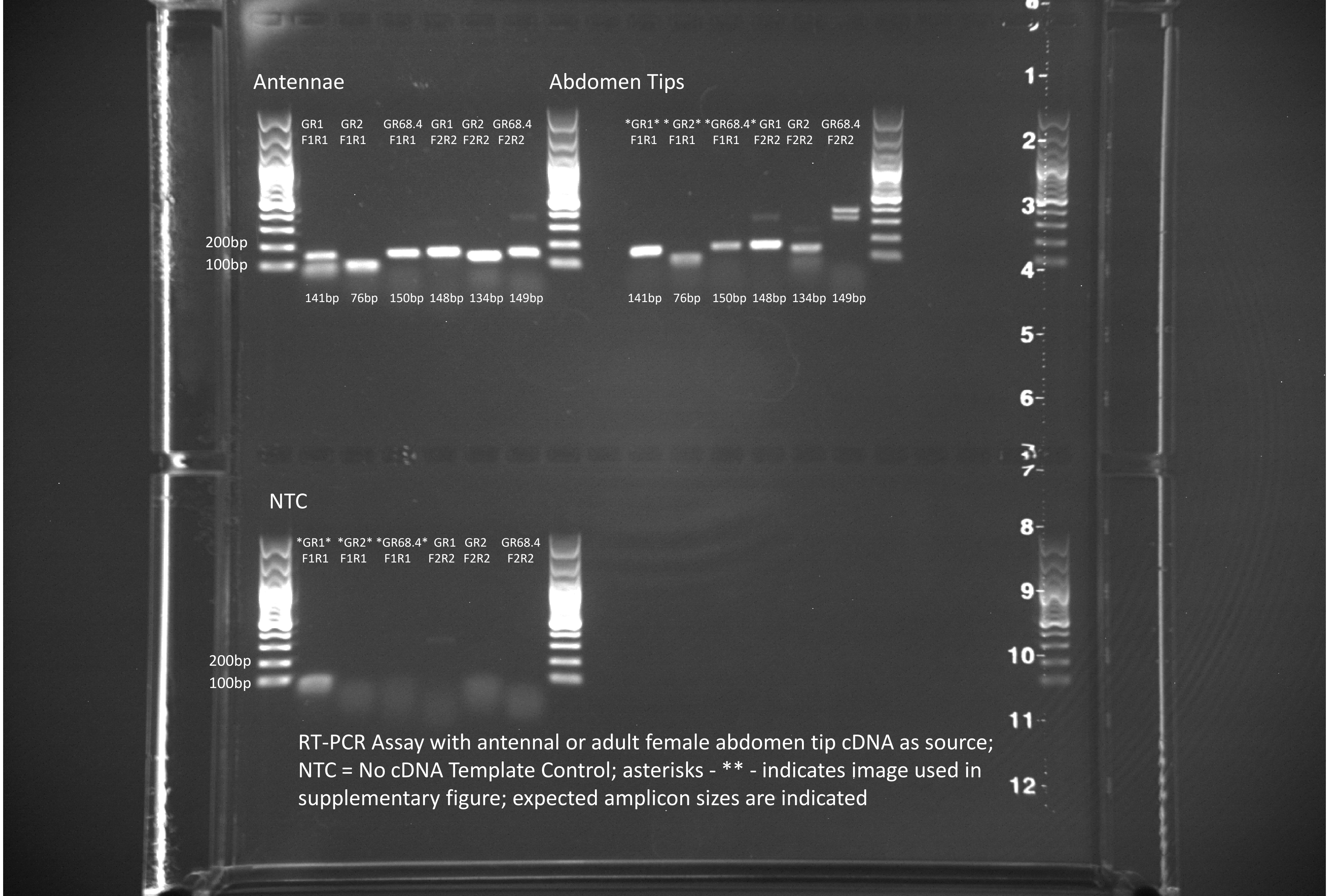
Antennae
Abdomen Tips
GR1 F1R1
GR2 F1R1
GR68.4 F1R1
GR1 F2R2
GR2 F2R2
GR68.4 F2R2
GR1 F1R1
* GR2* F1R1
GR68.4 F1R1
GR1 F2R2
GR2 F2R2
GR68.4 F2R2
200bp
100bp
141bp 76bp 150bp 148bp 134bp 149bp
141bp 76bp 150bp 148bp 134bp 149bp
NTC
GR1 F1R1
GR2 F1R1
GR68.4 F1R1
GR1 F2R2
GR2 F2R2
GR68.4 F2R2
200bp
100bp
1
2
3
4
5
6
7
8
9
10
11
12
RT-PCR Assay with antennal or adult female abdomen tip cDNA as source;
NTC = No cDNA Template Control; asterisks - ** - indicates image used in
supplementary figure; expected amplicon sizes are indicated

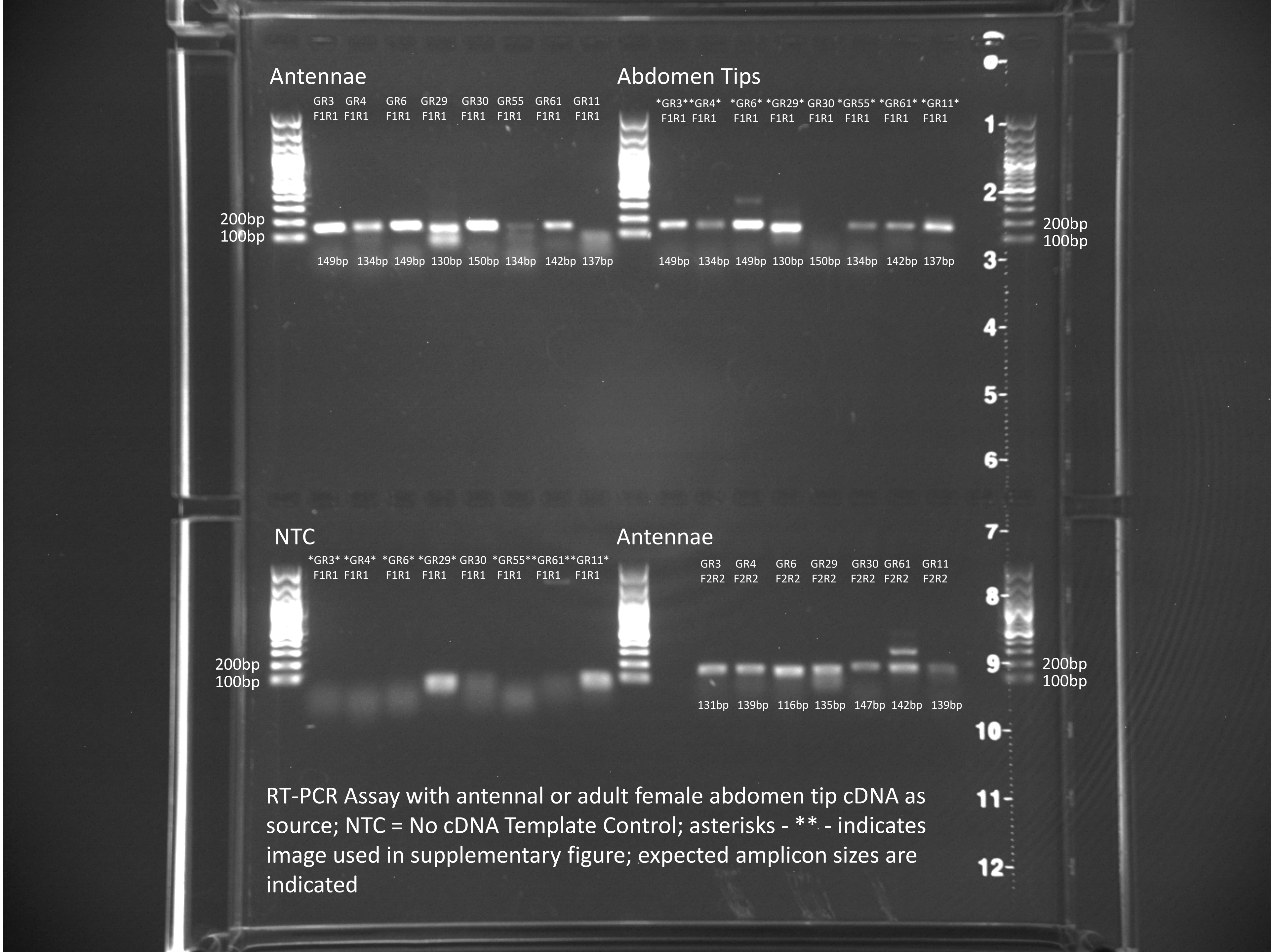

RT-PCR Assay with antennal or adult female abdomen tip cDNA as source; NTC = No cDNA Template Control; asterisks - ** - indicates image used in supplementary figure; expected amplicon sizes are indicated

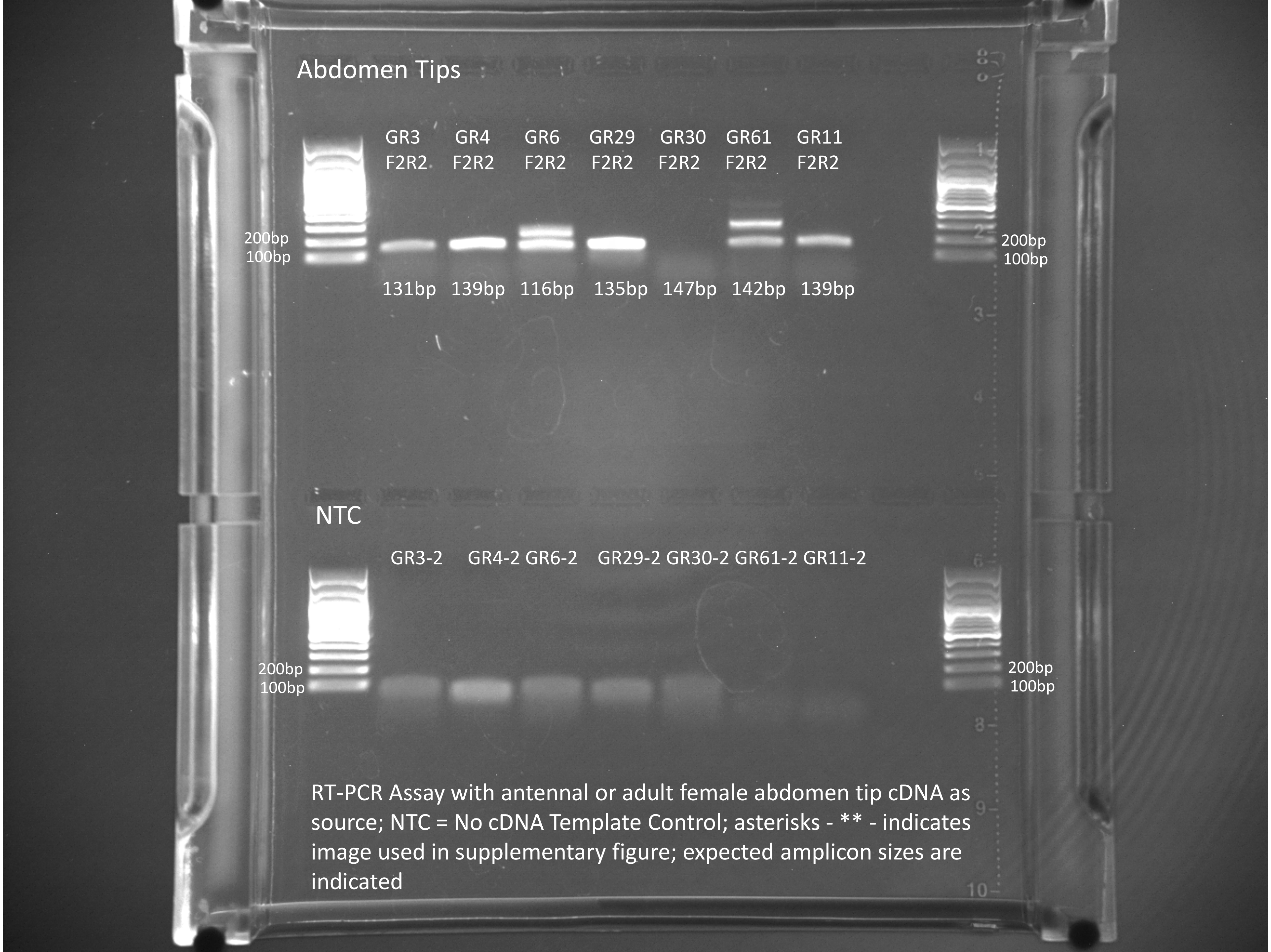

RT-PCR Assay with antennal or adult female abdomen tip cDNA as source; NTC = No cDNA Template Control; asterisks - ** - indicates image used in supplementary figure; expected amplicon sizes are indicated

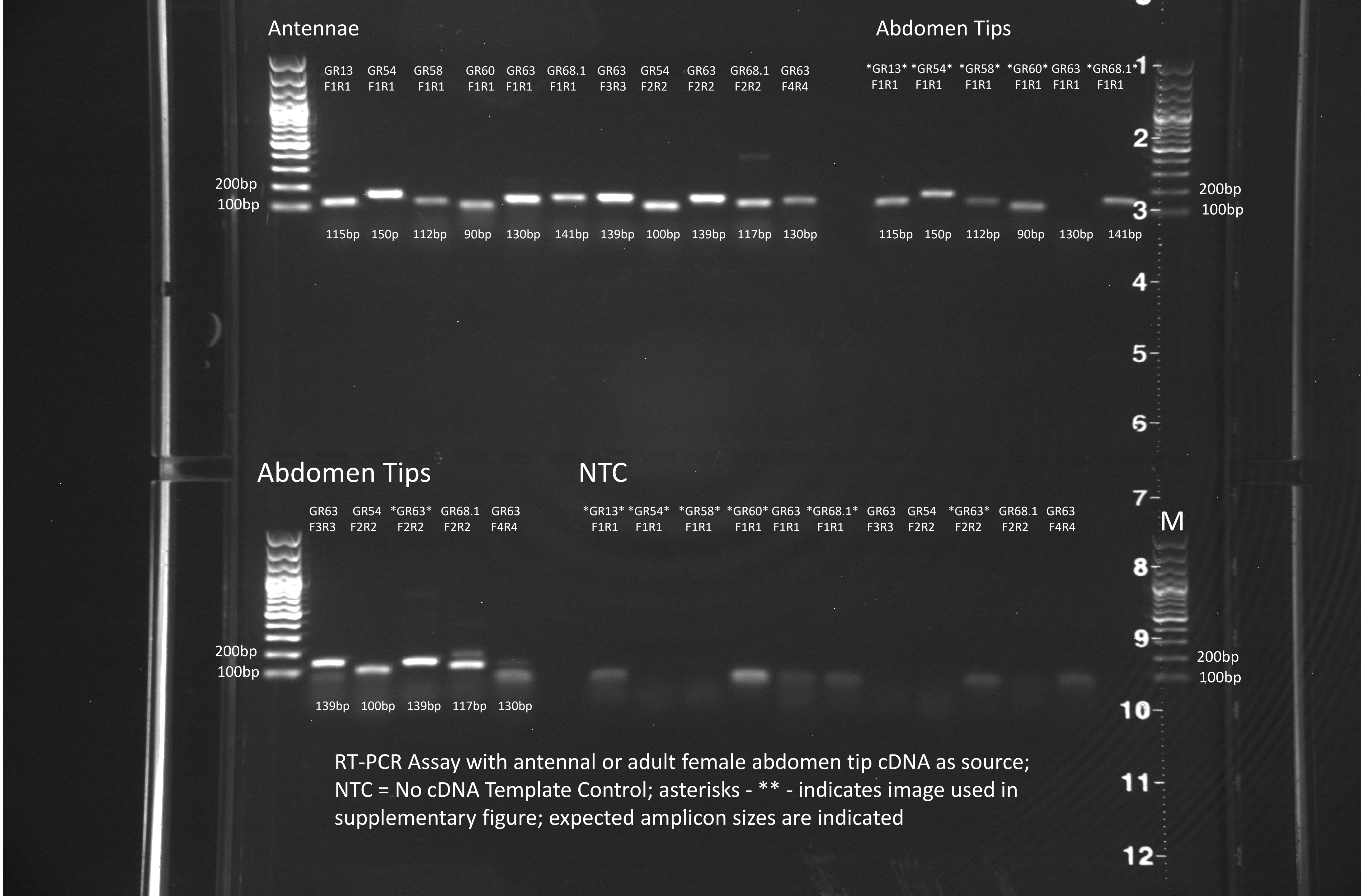

RT-PCR Assay with antennal or adult female abdomen tip cDNA as source; NTC = No cDNA Template Control; asterisks - ** - indicates image used in supplementary figure; expected amplicon sizes are indicated

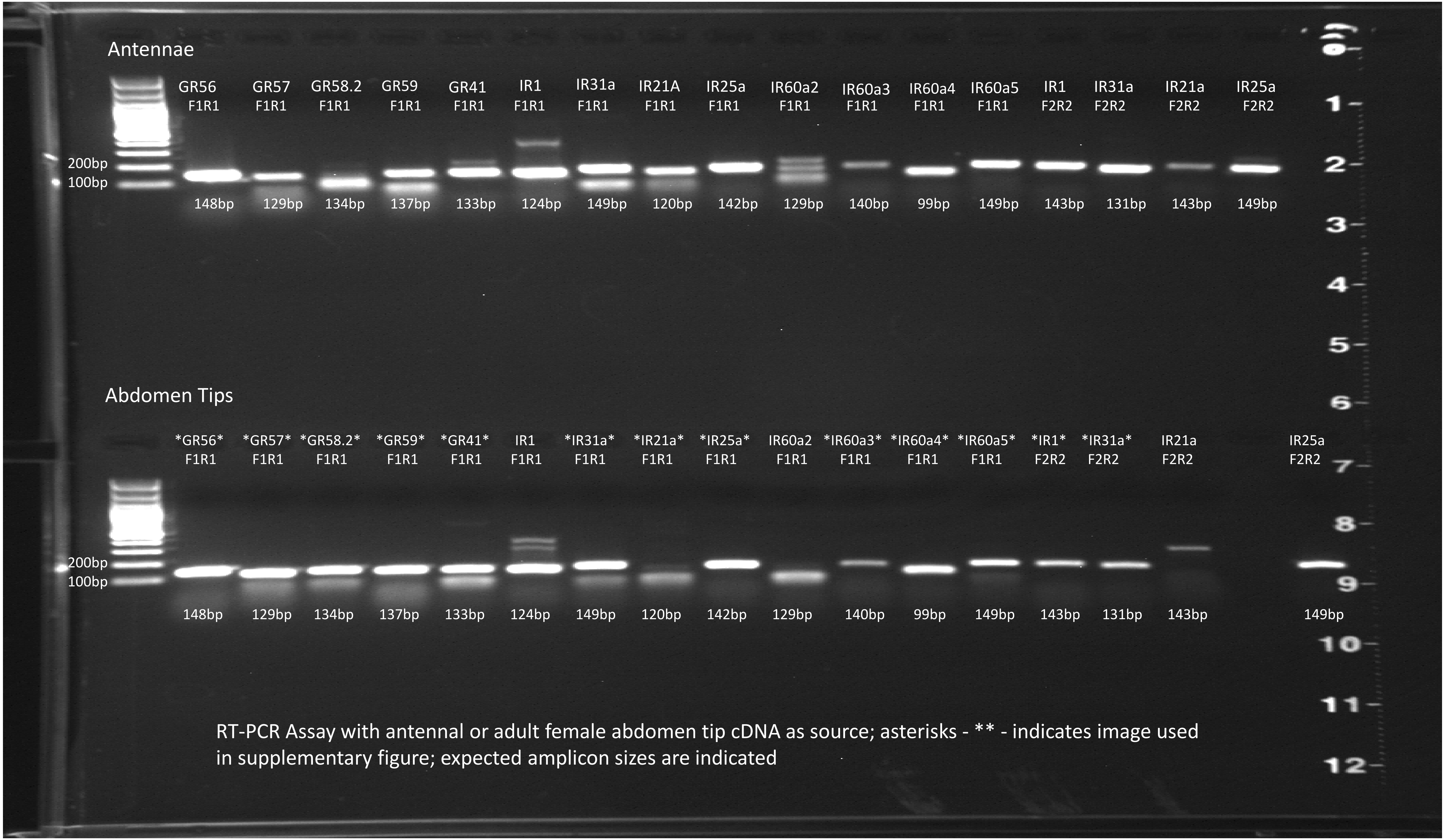

RT-PCR Assay with antennal or adult female abdomen tip cDNA as source; asterisks - ** - indicates image used in supplementary figure; expected amplicon sizes are indicated

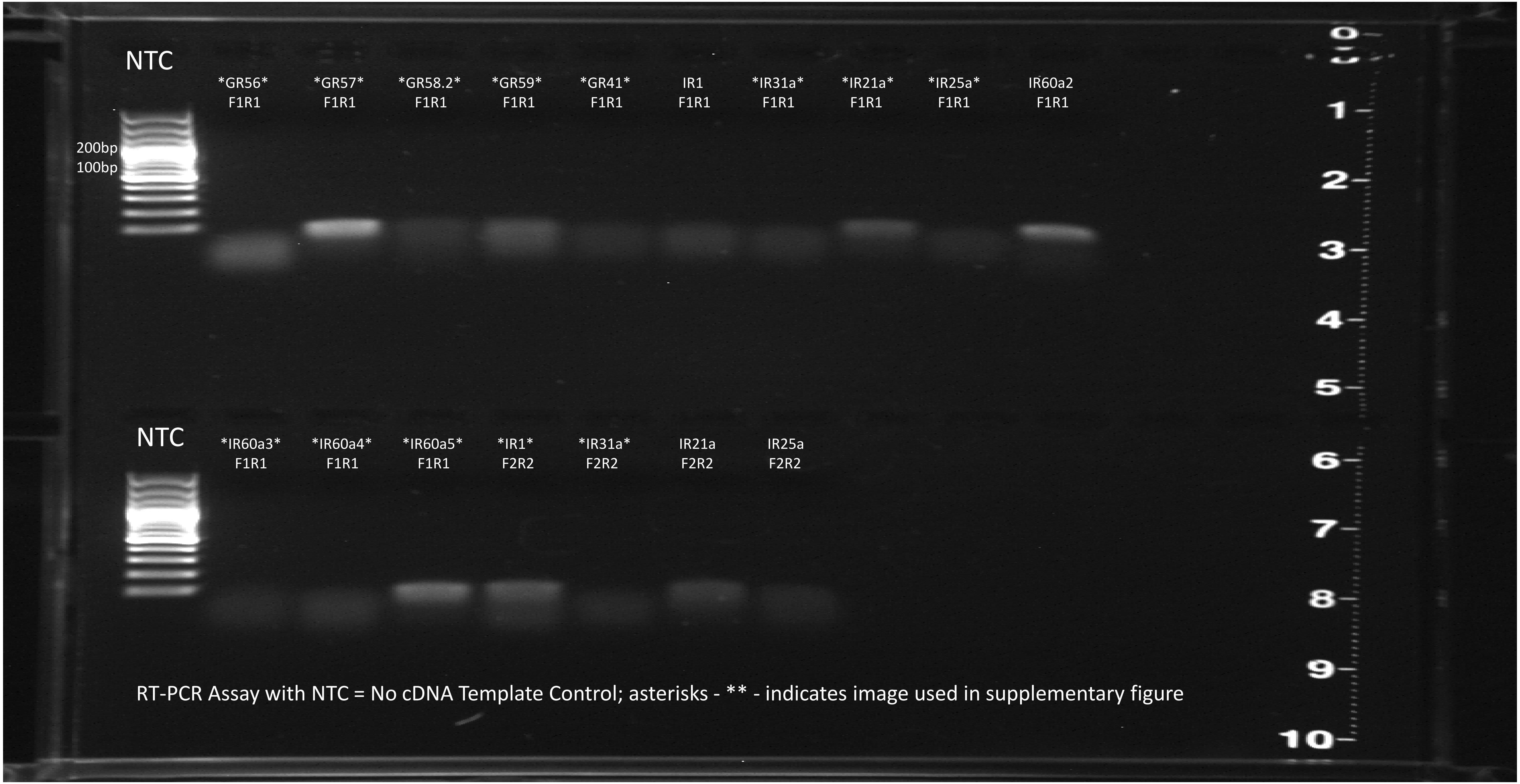

RT-PCR Assay with NTC = No cDNA Template Control; asterisks - ** - indicates image used in supplementary figure

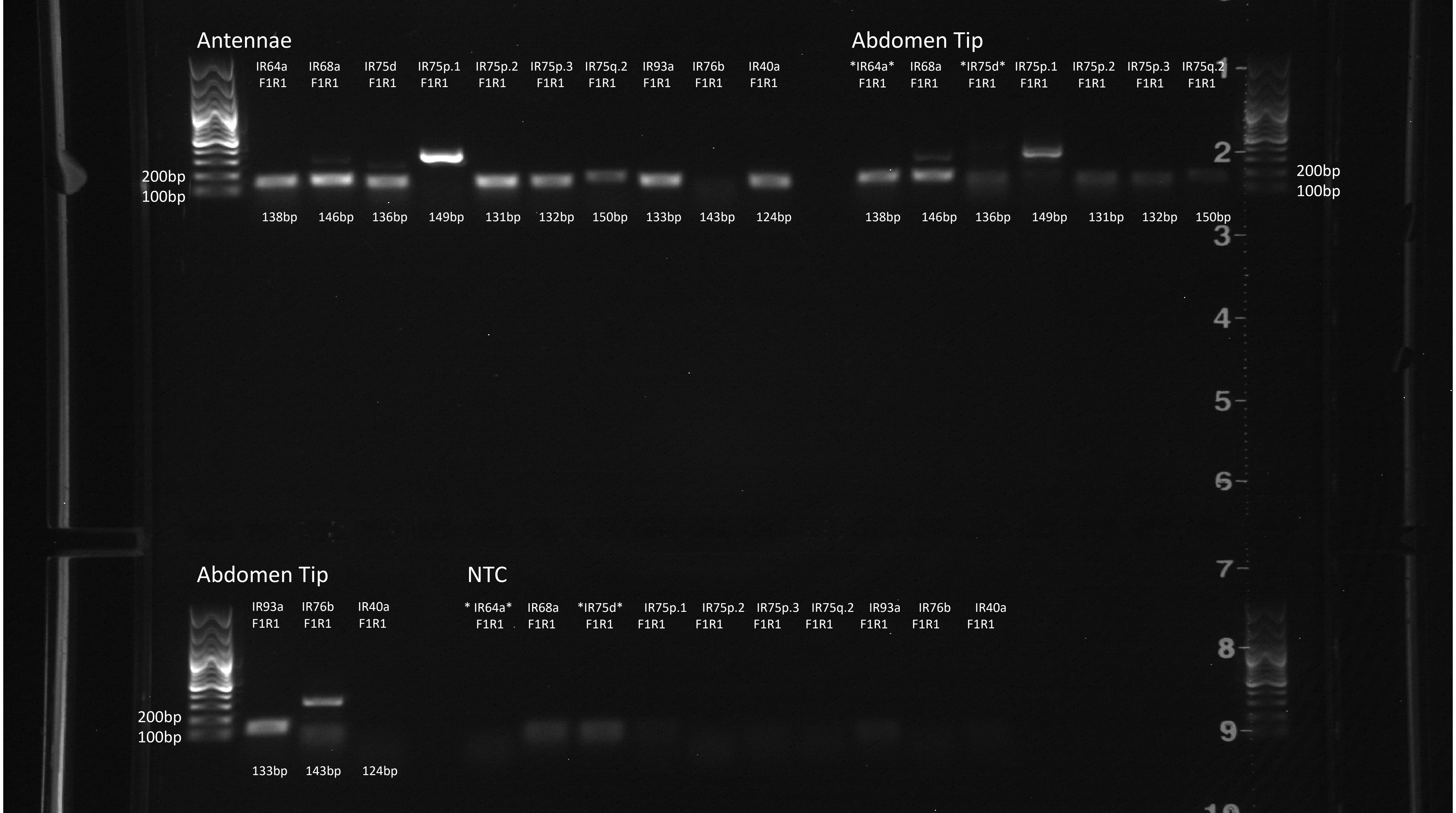

RT-PCR Assay with antennal or adult female abdomen tip cDNA as source; NTC = No cDNA Template Control; asterisks - ** - indicates image used in supplementary figure; expected amplicon sizes are indicated

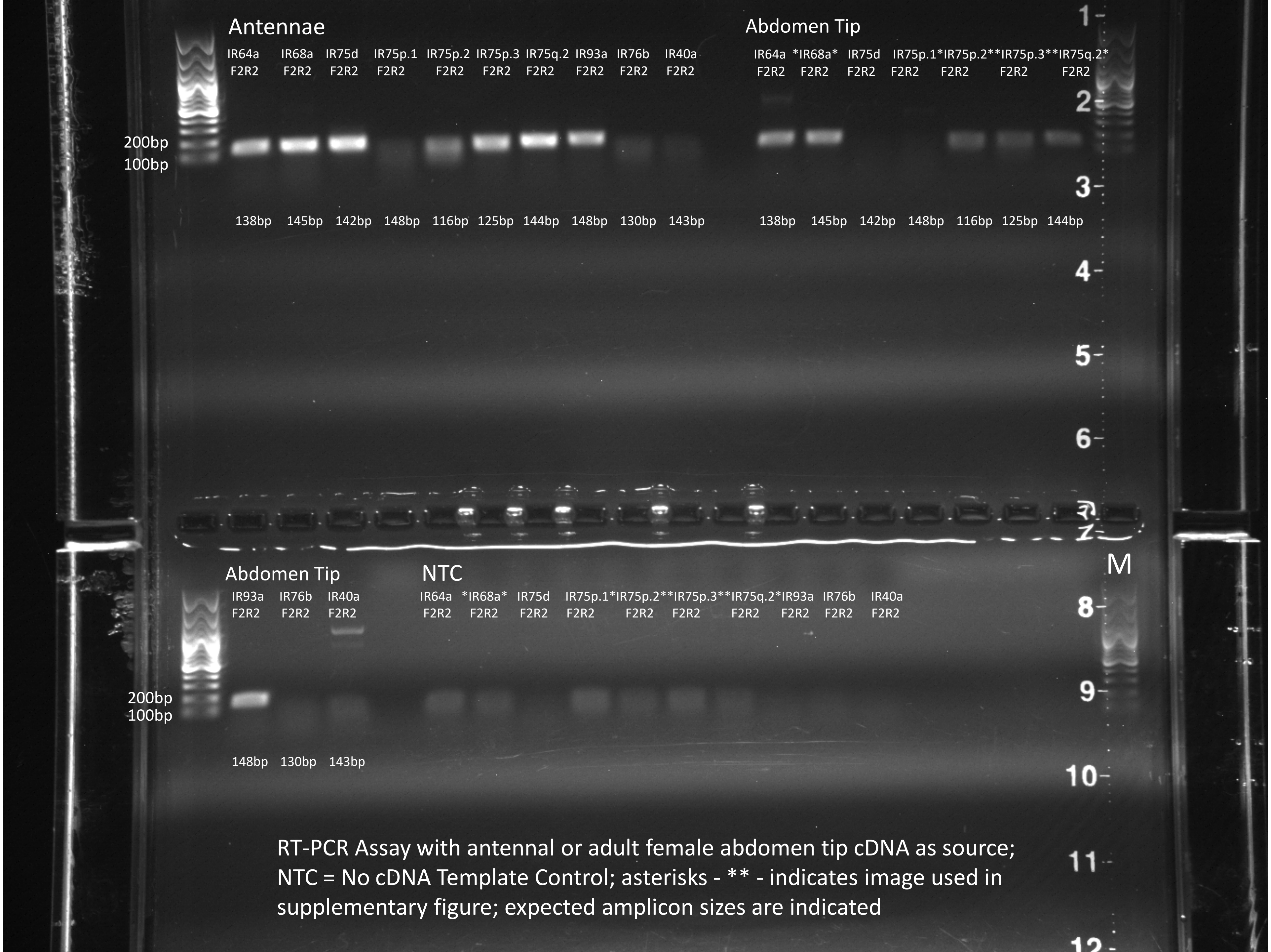

RT-PCR Assay with antennal or adult female abdomen tip cDNA as source; NTC = No cDNA Template Control; asterisks - ** - indicates image used in supplementary figure; expected amplicon sizes are indicated

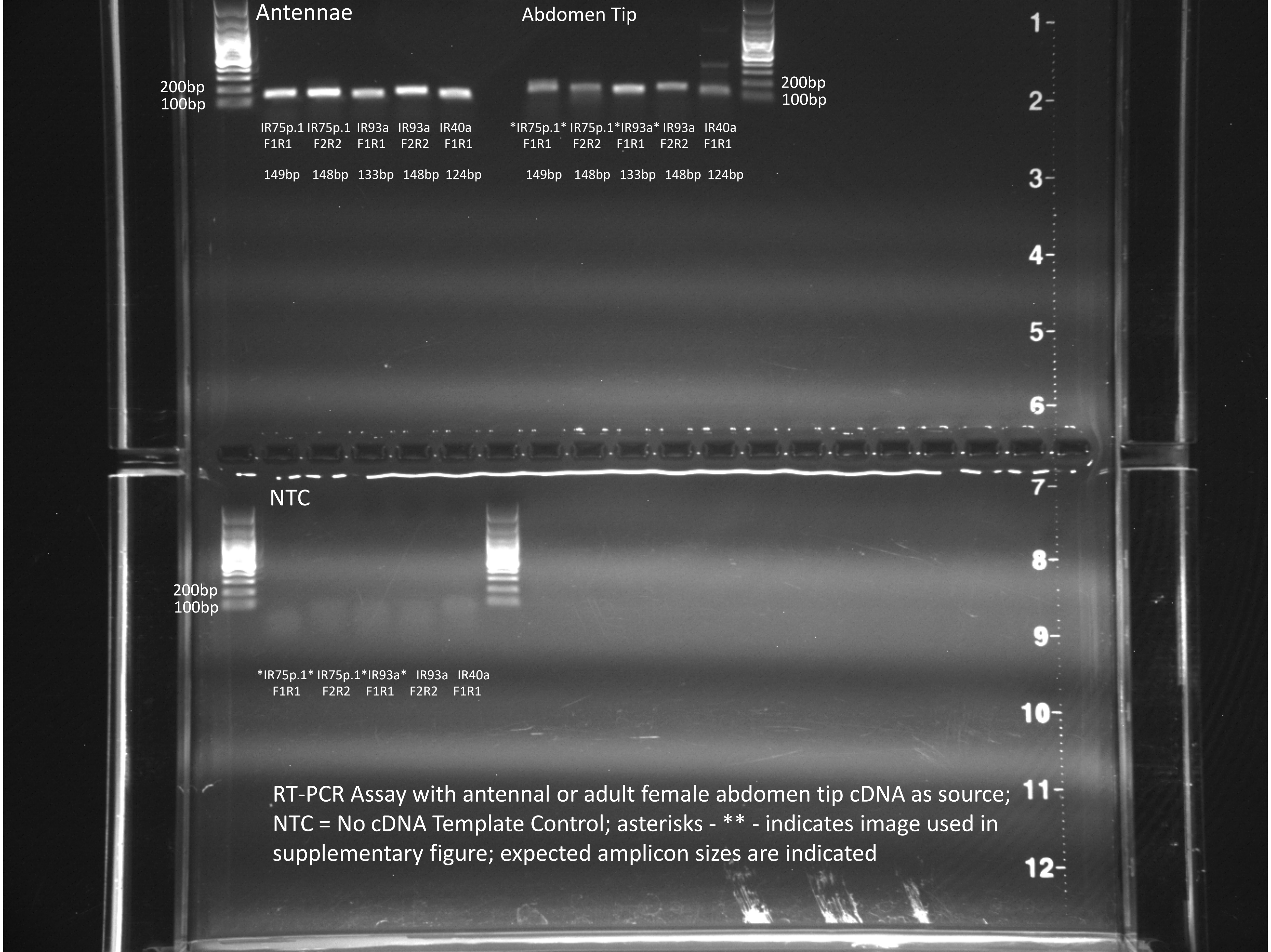

RT-PCR Assay with antennal or adult female abdomen tip cDNA as source; NTC = No cDNA Template Control; asterisks - ** - indicates image used in supplementary figure; expected amplicon sizes are indicated

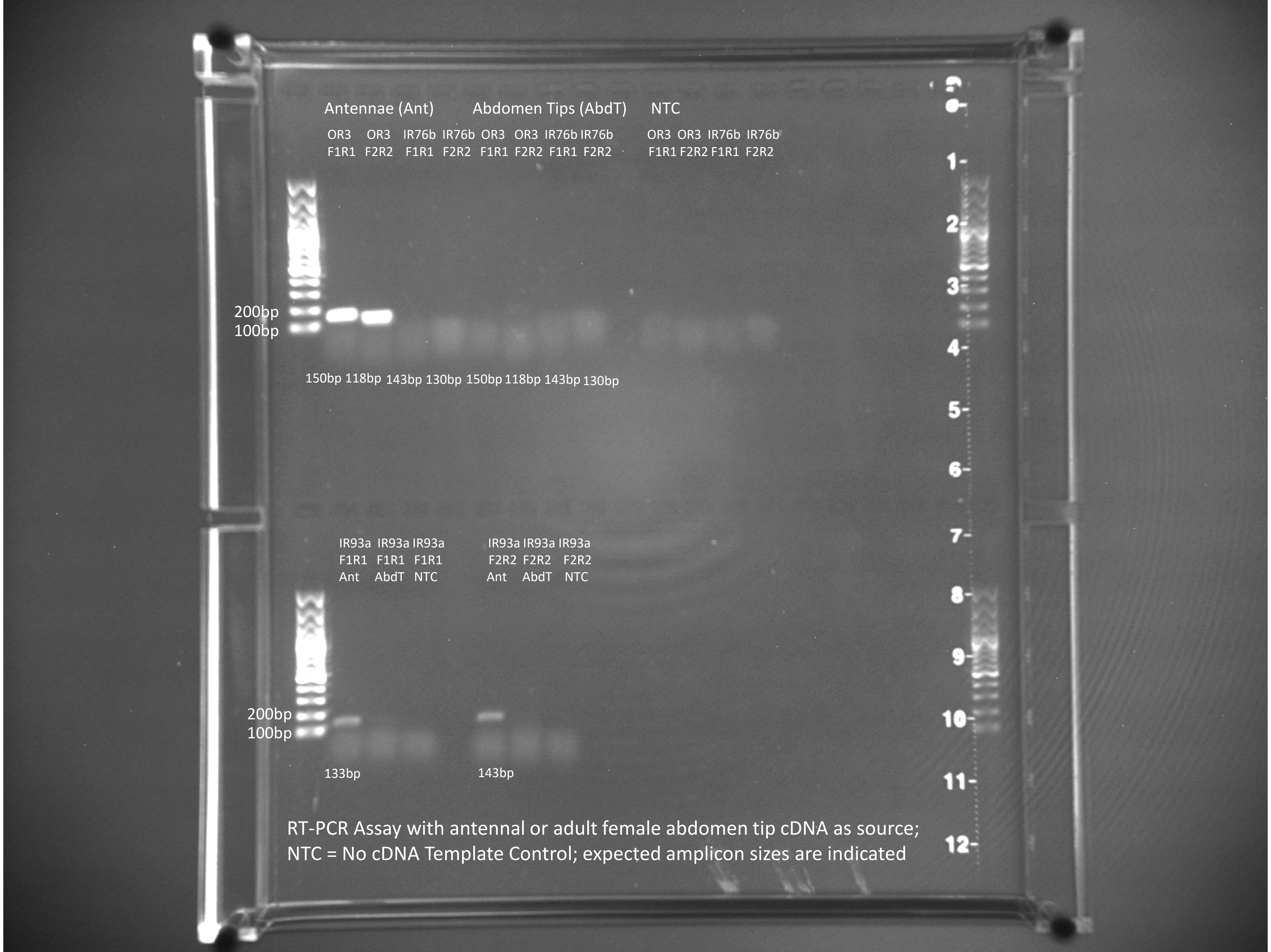

RT-PCR Assay with antennal or adult female abdomen tip cDNA as source;
NTC = No cDNA Template Control; expected amplicon sizes are indicated
