# Supplementary figures and images for: Chemosensory Receptor Expression in the Abdomen Tip of the Female Codling Moth, Cydia pomonella L. (Lepidoptera: Tortricidae)
Source: Insects. 2023 Dec 14;14(12):948. doi: 10.3390/insects14120948 (PMC10743790; doi:10.3390/insects14120948)

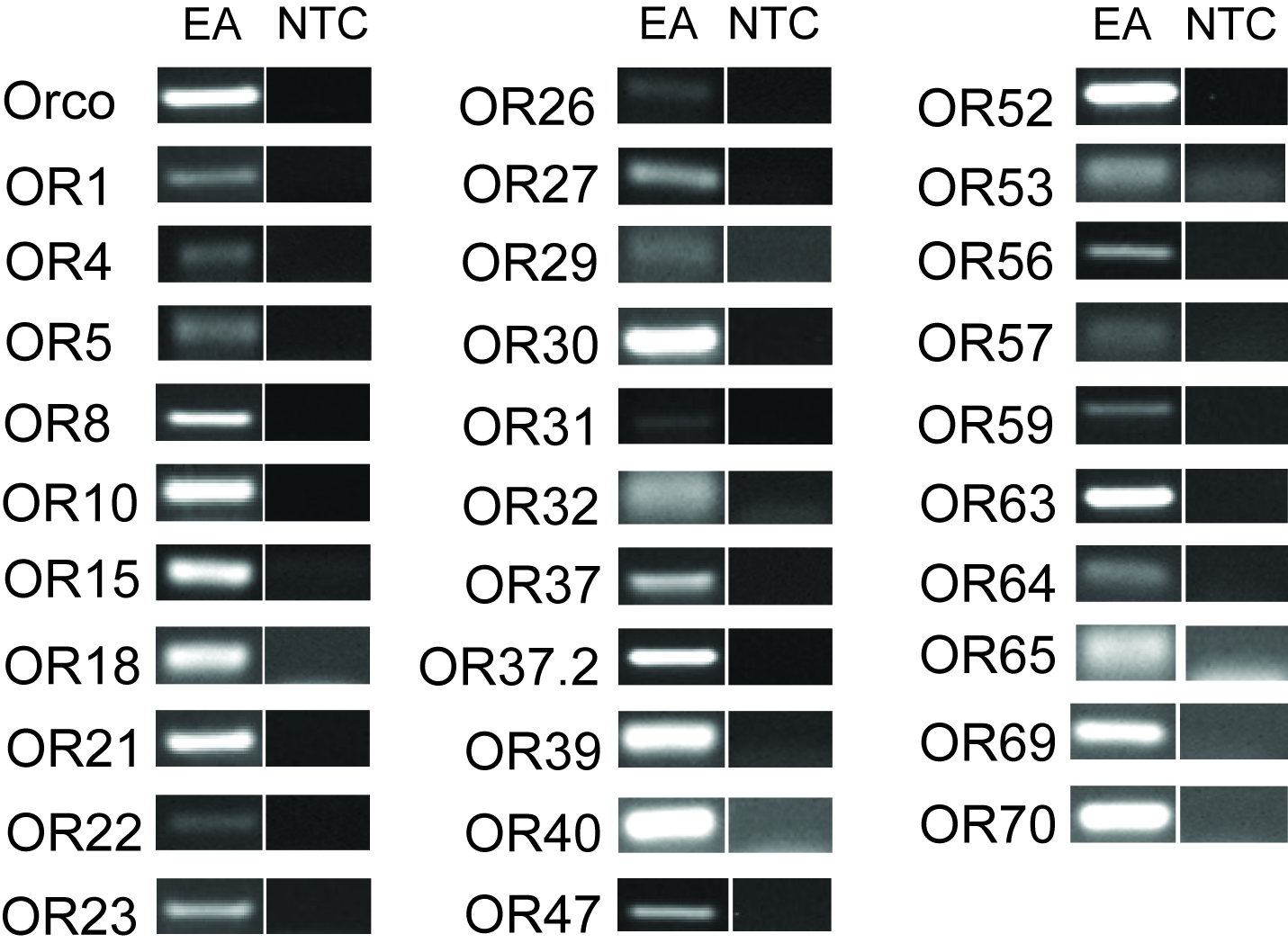

Supplement: Supplementary file 1 [file insects-14-00948-s001.zip › Figure S1 - RTPCR Assay of OR Expression_final.tif]

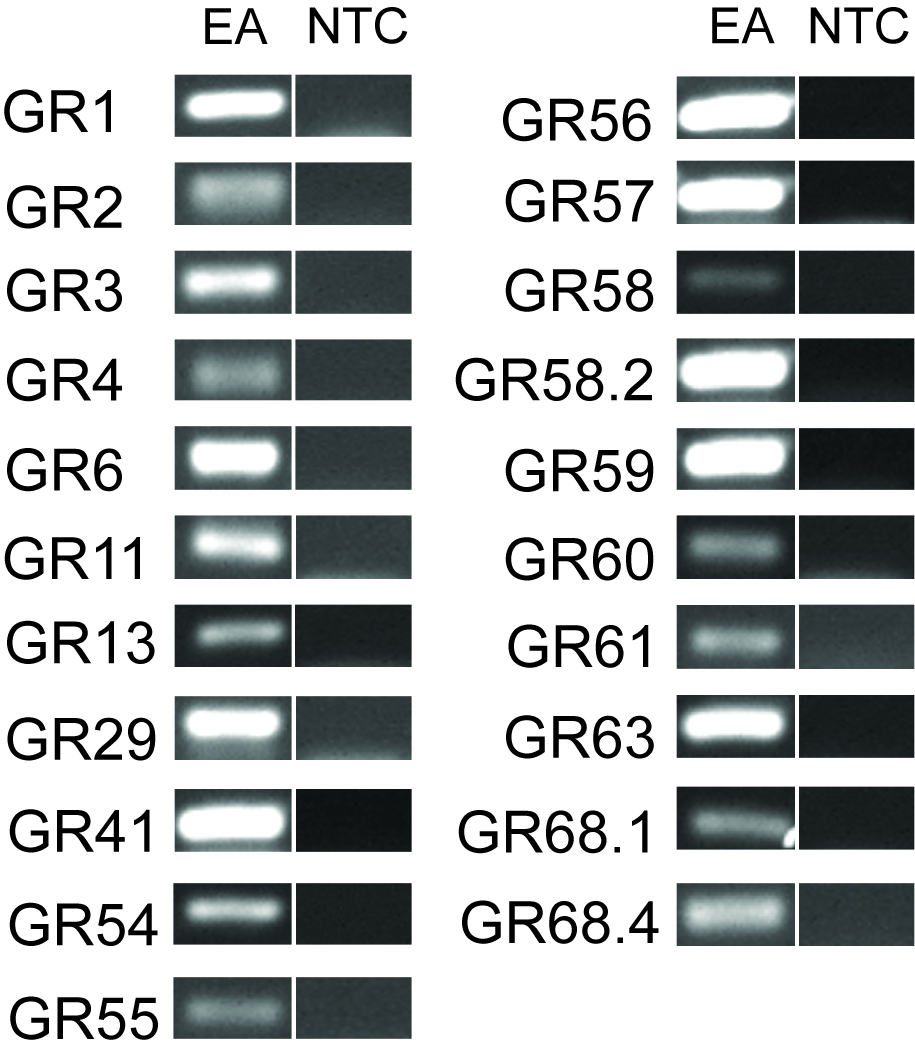

Supplement: Supplementary file 1 [file insects-14-00948-s001.zip › Figure S2 - RTPCR Assay of GR Expression_final.tif]

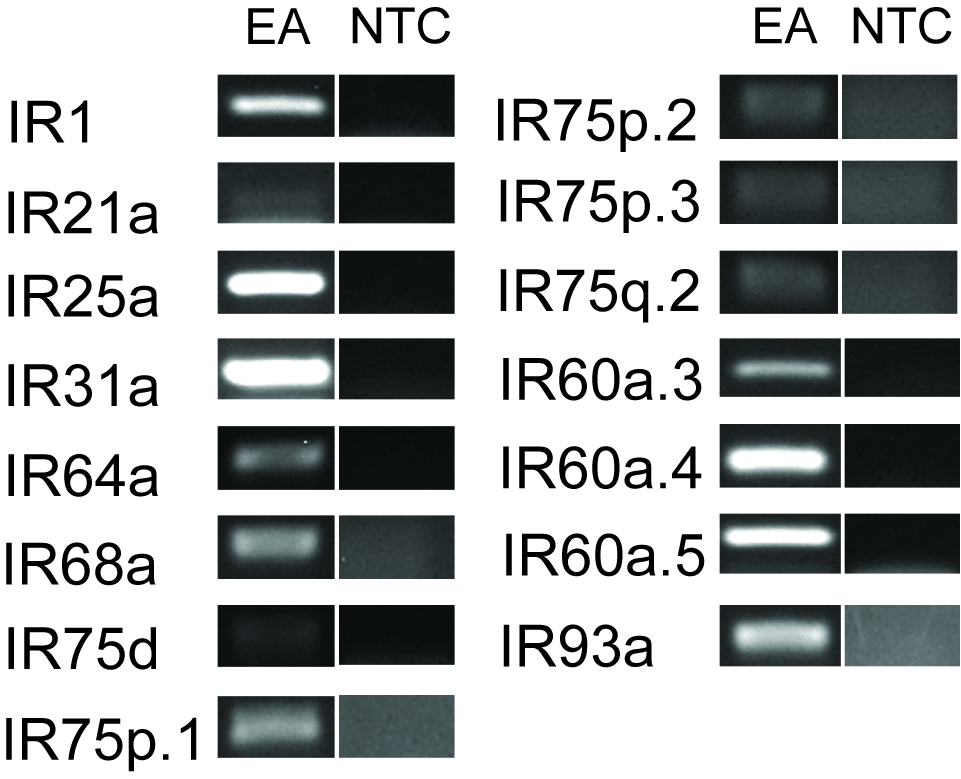

Supplement: Supplementary file 1 [file insects-14-00948-s001.zip › Figure S4 - RTPCR Assay of IR Expression_final.tif]
