# Supplementary material for: Chemosensory Receptor Expression in the Abdomen Tip of the Female Codling Moth, Cydia pomonella L. (Lepidoptera: Tortricidae)
Source: Insects. 2023 Dec 14;14(12):948. doi: 10.3390/insects14120948 (PMC10743790; doi:10.3390/insects14120948)

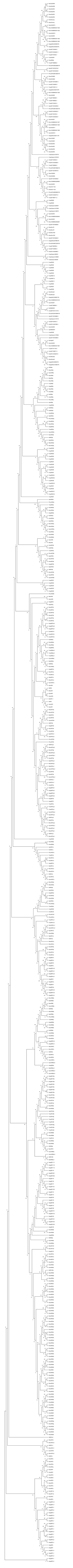

Supplement: Supplementary file 1 [file Figure S3 - MegaBroadIRs Phylogeny.pdf › Figure S3 - MegaBroadIRs Phylogeny.pdf]
